# Transmission Electron Microscopy Corneal Ultrastructure Study in Hematocornea of Corneal Transplant Graft

**DOI:** 10.3390/diagnostics16060890

**Published:** 2026-03-17

**Authors:** Paul Filip Curcă, Laura Macovei, Ovidiu Mușat, Mihail Zemba, Valentin Dinu, Mihaela Gherghiceanu, Cătălina Ioana Tătaru, Călin Petru Tătaru

**Affiliations:** 1 Department of Ophthalmology, “Dr. Carol Davila” Central Military Emergency University Hospital, Carol Davila University of Medicine and Pharmacy, 010825 Bucharest, Romania; filipcurca@yahoo.com (P.F.C.); laura.macovei@umfcd.ro (L.M.); mhlzmb@yahoo.com (M.Z.); calinpetrutataru@yahoo.com (C.P.T.); 2 Department of Ophthalmology, Clinical Institute for Ophthalmological Emergencies “Prof. Dr. Mircea Olteanu”, Carol Davila University of Medicine and Pharmacy, 010464 Bucharest, Romania; valentin.dinu@umfcd.ro (V.D.); catalina_tataru@yahoo.com (C.I.T.); 3 Victor Babeș National Institute for Pathology, 050096 Bucharest, Romania; mihaela.gherghiceanu@umfcd.ro; 4 Department of Ophthalmology, Clinical Institute for Ophthalmological Emergencies “Prof. Dr. Mircea Olteanu”, 010464 Bucharest, Romania

**Keywords:** hematocornea, corneal blood staining, transmission electron microscopy, TEM, corneal transplant, deep anterior lamellar keratoplasty, DALK, corneal graft

## Abstract

**Background and Clinical Significance**: To our knowledge, there is a lack of electron microscopy studies in hematocornea since 1985, and more so for graft hematocornea after deep anterior lamellar keratoplasty (DALK). This study provides an ultrastructural characterization of hematocornea occurring in a DALK graft. Our study presents several limitations: single-case design and lack of control tissue. **Case Presentation**: The DALK graft with hematocornea was excised and introduced inside of the operating room in glutaraldehyde solution recipient. The graft was quickly cold-transported to light and transmission electron microscopy. Hematocornea in the DALK transplant graft resulted in features of stromal alteration and dysfunctional cellular clean-up response. The collagen lamellae ultrastructure was affected near electron-dense hem deposits. Two cellular aspects were observed: adaptation and degeneration. Electron-dense granules were found in keratocytes, which may exhibit cellular adaptations, such as vacuoles and phagosomes. Macropinocytosis may mechanistically explain ingestion of electron-dense granules, and dysfunctions in the macropinocytosis process may have led to cell degeneration. Cellular degeneration was marked by loss of organelle contour and loss of cellular membrane integrity (burst-cell aspect). Microscopic corneal alteration corresponded to macroscopic total loss of corneal transparency and elasticity. **Conclusions**: This study described lamellar ultrastructure alterations and dysfunctional cellular response in hematocornea of a DALK corneal transplant graft.

## 1. Introduction

### 1.1. Deep Anterior Lamellar Keratoplasty (DALK)

Deep anterior lamellar keratoplasty (DALK) is the replacement of pathological corneal stroma with a donor graft while sparing the two inner corneal layers of Descemet’s membrane and the endothelium [1,2]. Preservation of healthy corneal endothelium is advantageous. DALK preserves endothelial cell count and reduces overall complication rates compared to full-corneal-thickness penetrative keratoplasty [1,2]. Furthermore, overall graft rejection risk is reduced [1,2]. DALK is associated with several postoperative complications, most frequently Descemet’s membrane perforation [2,3], secondary glaucoma [2,3], cataract induction [2,3], suture-related complications, and graft rejection [2,3]. Descemet’s membrane serves as a barrier between the patient’s endothelium and the graft’s stroma. Microscopic (<0.5 mm) [4] or macroscopic intraoperative perforations of Descemet’s membrane can produce an unstable anterior chamber (AC) or persistent collapse of the AC [4]. These, in turn, can produce further cascading iatrogenic complications. If perforation of Descemet’s membrane occurs, it is recommended to adapt the technique and continue DALK surgery as long-term outcomes are better versus conversion to traditional penetrative keratoplasty [4,5]. In particular, peripheral Descemet’s ruptures can often be successfully managed [6], and conversion to penetrative keratoplasty should be avoided, if possible, in these cases [6]. DALK rescue techniques can include lowering intraocular pressure via paracentesis [7], intracameral air tamponade to restore the anterior chamber [7], or anchoring sutures [8].

### 1.2. DALK and Hematocornea

Hematocornea is a condition distinguished by blood accumulation within the cornea [9]. Iatrogenic hematocornea can occur following previous intraocular surgery, wherein after a hemorrhage into the anterior chamber blood products pass through a damaged part of Descemet’s membrane, allowing hem products to pool between the Descemet and pre-Descemet’s membranes [9]. According to Kodavoor et al., breach of Descemet’s membrane is the most frequent complication associated with DALK (6.54–6.6% of cases) [2,3]. The intraoperative breach of Descemet’s membrane thus differentiates iatrogenic hematocornea from simple blood staining where blood slowly diffuses from an anterior chamber hemorrhage through an intact Descemet’s membrane [9]. Clearence of hemoglobin products is slow through diffusion [9,10] with oxyhemoglobin the preponderant clearance product [11], followed by a gradual increase in the proportion of methemoglobin [11] and degradation to bile products [10].

### 1.3. Previous Transmission Electron Microscopy (TEM) Studies in Hematocornea

To date, a few studies have been published on electron microscopy in hematocornea.

McDonnell, P. J. et al. [10] noted that Pouliquen and Desvignes first described ultrastructural aspects in hematocornea [10] such as relatively intact stromal lamellae and numerous large deposits of electron-dense products from erythrocyte degradation inside of keratocytes and throughout the extracellular matrix [10].

Yoshimura M. et al. described ultrastructural aspects of intracorneal hemorrhage in interstitial keratitis due to Hansen’s disease [12], finding scar formation and disarranged and destroyed lamellae with electron-dense granules of irregular or rod-like shape between the lamellae [12]. Granules were also present in the cytoplasm of parenchymal cells, either enveloped by thin membranes or inside of vacuoles [12]. The authors reported the frequent presence of cells regarded as macrophages, larger in size with abundant granules in the cytoplasm, as well as the presence of neovascular features [12].

McDonnell, P. J. et al. presented a larger electron microscopy study from a series of 11 corneal buttons and globes with hematocornea [10]. Eosinophilic electron-dense deposits were present throughout the stroma, with hemoglobin accumulations primarily located within stromal lamellae [10]. Keratocytes presented extensive vacuoles and degeneration [10]. Fine granules of hemosiderin were accumulated within epithelial cells and apparent hemoglobin globules between epithelial cells [10].

Messmer EP. et al. noted that moderately electron-dense, amorphous material presumed to be hemoglobin was found extracellularly, as well as incorporated within stromal keratocytes, some with necrosis, as well as electron-dense intracellular membrane-bound granules [13].

To date, this paper’s authors do not have knowledge of more recent electron microscopy studies in hematocornea. Furthermore, there is a lack of TEM studies upon DALK corneal transplant grafts with hematocornea.

### 1.4. The Corneal Ultrastructure

The integrity of the corneal ultrastructure is essential to corneal function and properties. The principal protein in the cornea is collagen, which is condensed into narrow uniform-diameter fibrils 30 nanometers wide [14]. The fibrils are organized into larger collagen lamellae with length up to 0.2 mm and thickness up to 2 microns [14,15]. Proteoglycan molecules maintain a short-range packing order of the collagen fibrils [14], and proteoglycan-bridging dimers allow for dynamic local movement of adjacent fibrils [14].

The corneal ultrastructure presents different lamellae directions. Anteriorly, lamellae insert at an angle with Bowman’s membrane [14]. The lamellae are interweaved in the immediate anterior stroma, supporting corneal curvature like interweaving fibers in a bird’s nest [14]. In the posterior two-thirds of the stroma, the lamellae lie over-imposed one on another and run from limbus to limbus like plywood [14]. In plywood, cross-graining provides high stiffness perpendicular to the grain direction and reduces overall warping; in the cornea, more varied lamellae directions provide strength in multiple directional axes. Finally, according to X-ray diffraction studies, the corneal annulus presents a dense collagen structure, serving as high-strength annular reinforcement for the cornea [14,15].

Corneal transparency is a consequence of a physiological balance of light scattering. The short-range order of fibrils counteracts light scattering (Rayleigh effect) [14] by producing constructive interference of all scattered waves in the forward direction and destructive interference in other directions [14]. Corneal stromal cells (keratocytes, 17% of stromal volume) have a similar refractive index to the stroma (1.381 ± 0.004), thus eliminating any cellular light scatter [14]. This physiological balance of light scattering assures corneal transparency and optical performance.

## 2. Materials and Methods

### 2.1. Clinical Context of Case Presentation

**Figure 1:**
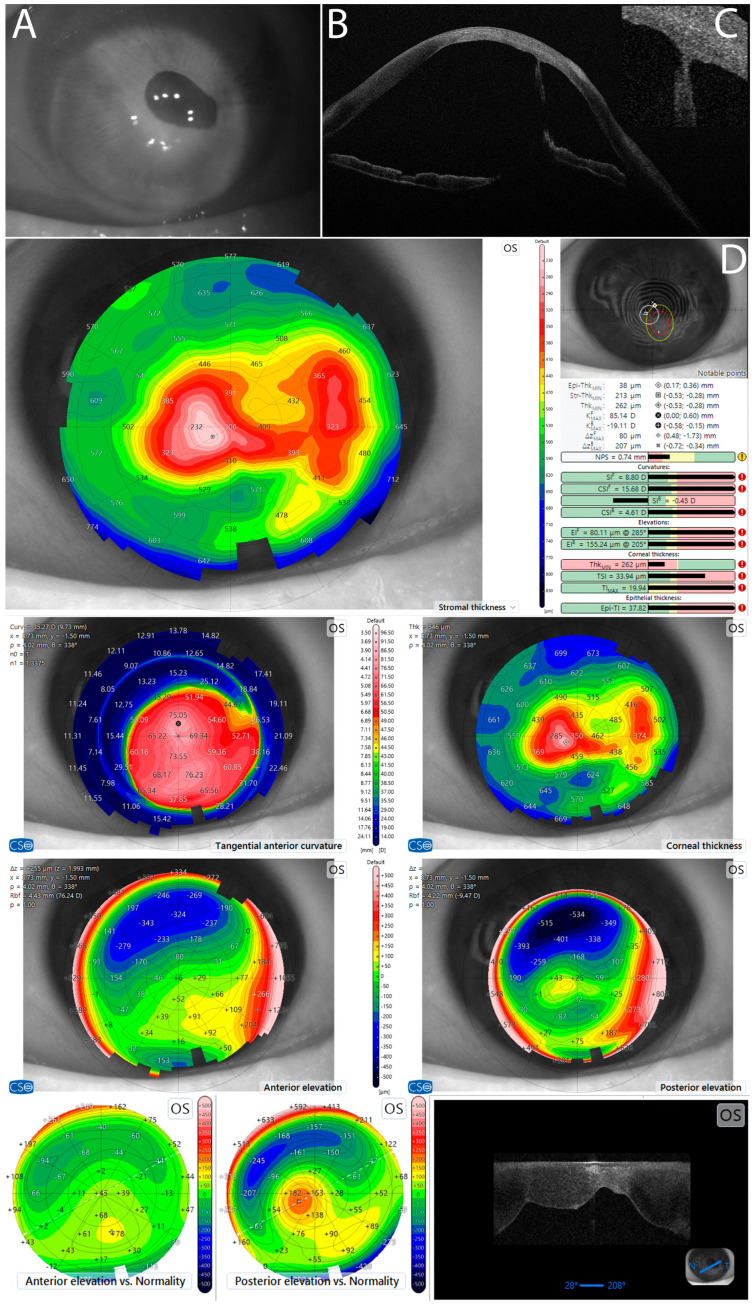
Presentation with post-traumatic secondary corneal ectasia. (**A**,**B**) Corneal–iris adherence with iris displaced upwards. (**C**) Possible compromise of Descemet’s membrane integrity (adherence site). (**D**) Corneal data and tomography (AS-OCT MS-39, CSO, Scandicci, Italy).

**Figure 2:**
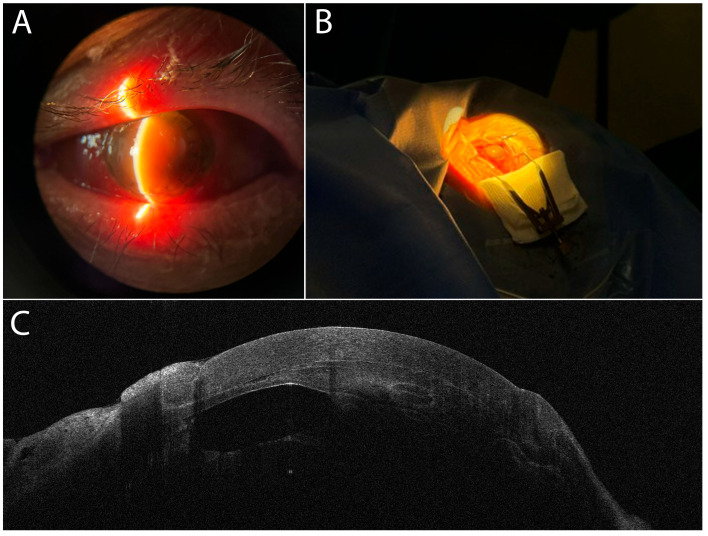
Presentation with hematocornea after previous DALK. (**A**) Slit-lamp examination: brownish-yellow corneal opacity, hyphema, functional DALK graft. (**B**) Preoperative macroscopic aspect: loss of graft transparency. (**C**) AS-OCT image: blood clots and adherences in AC; demarcation of iris tissue is unclear. (AS-OCT MS-39, CSO, Scandicci, Italy).

The patient, with known history of cataract surgery, initially presented for post-traumatic secondary corneal ectasia. Clinical examination and combined placido disc topography and high-resolution anterior segment optical coherence (AS-OCT) tomography (AS-OCT MS-39, CSO, Scandicci, Italy) revealed high-risk corneal ectasia (Figure 1). Post-traumatic corneal–iris adherence was also noted, with the iris displaced upwards (Figure 1A,B). It is possible that integrity of Descemet’s membrane was compromised at the site of this adherence (Figure 1C). Treatment with DALK was performed. During DALK, we observed iatrogenic breach of Descemet’s membrane, followed quickly by anterior chamber (AC) collapse. To restore the AC, an inferior peripheral iridotomy was performed; however, this maneuver produced a severe and unexpected intracameral hemorrhage, which was difficult to control. Postoperative treatment focused on hemostatic agents and inflammation control. One month after DALK, the patient presented hand-motion visual acuity (VA). Slit-lamp examination revealed hematocornea (Figure 2), considered a cascading iatrogenic complication of the previous DALK Surgery. 

Due to iatrogenic breach of Descemet’s membrane, the blood pooled in the anterior chamber from the iridectomy could more easily infiltrate the DALK graft, producing the resultant hematocornea.

**Figure 3:**
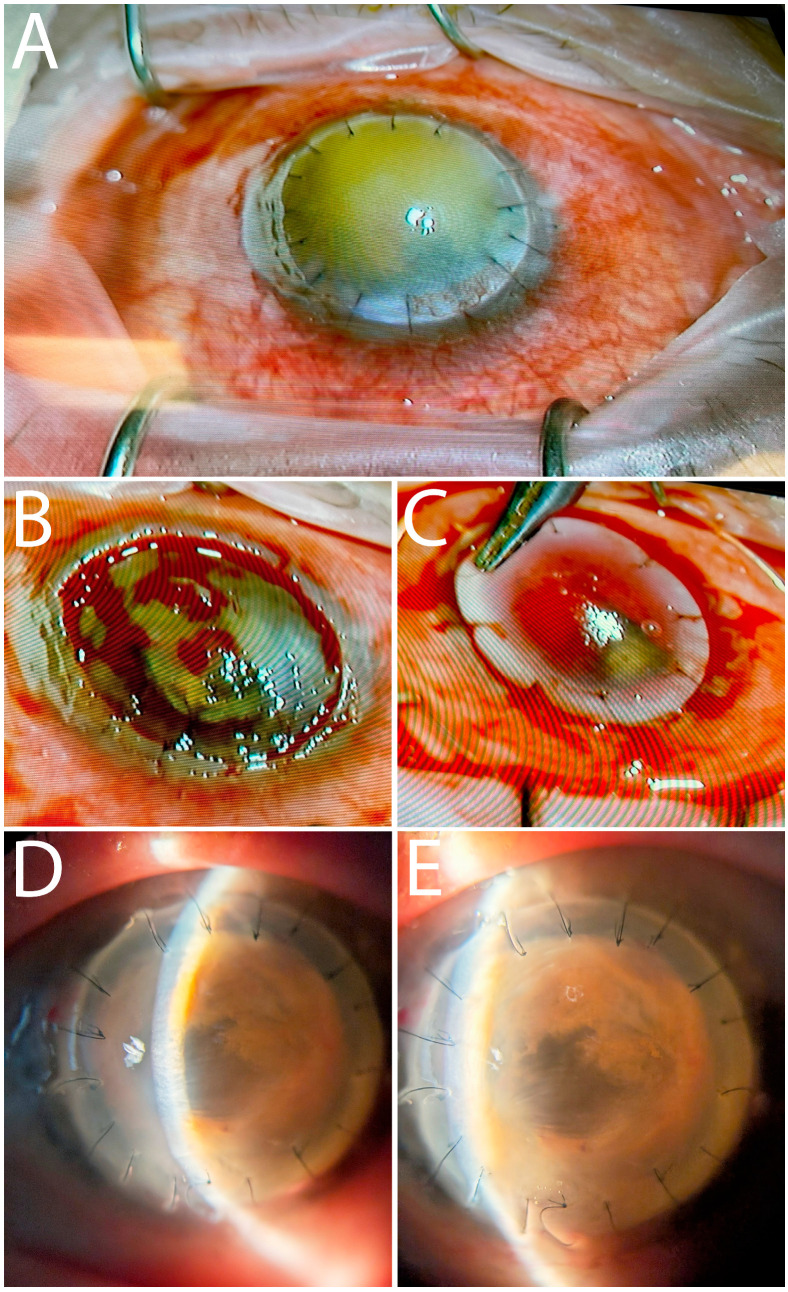
(**A**) Intraoperative aspect of the hematocornea DALK transplant graft: brownish-yellow opacity. (**B**) Underlying extensive blood clots and iris tissue adherences; IOL entrapped in adherences. (**C**) Suture placement after performing PK; easy bleeding from iris adherences. (**D**,**E**) Postoperative 4-month follow-up. Restored AC, clear visual axis, no resurgence of hyphema or inflammation. Several sutures were removed at this follow-up. BCVA improved to 20/100–20/200 (patient aphakic).

After having been provided with informed patient consent, the surgical team performed a penetrating keratoplasty re-transplant in the patient (Figure 3). Intraoperatively, under the hematocornea, extensive blood clots and iris tissue adherences were encountered (Figure 3B). Synechiolysis, excision of blood clots, and explant of the patient’s IOL, which was displaced and entrapped between adherences, were performed. The patient’s re-transplant procedure was successful in restoring anterior chamber depth, a clear visual axis, and improving best corrected visual acuity (BCVA) to 20/100–20/200 (patient aphakic). The patient remains in good follow-up without resurgence of hyphema or intraocular inflammation (Figure 3D,E).

### 2.2. Sample Preparation and Analysis

The hematocornea-stained graft (Figure 3A) was excised and immediately introduced inside of the operating-room in glutaraldehyde solution recipient. The sample was quickly cold-transported to the institute for electron microscopy (20 min in transport).

For light microscopy, the samples were colored using toluidine blue, and the images were obtained using a Leica optical microscope with a Leica DFC7000T digital color camera (Leica Microsystems, Wetzlar, Germany).

For transmission electron microscopy (TEM), small corneal fragments were fixed using immersion in glutaraldehyde 4% with buffer of sodium cacodylate (Table 1). Post-fixation was performed using osmium tetroxide 1% with sodium ferrocyanide (Table 1). After successive dehydrations using increasingly concentrated alcohol solutions and propylene oxide (Table 1), the tissue samples were included in epoxy resin (Agar 100). It is important to note that according to synchrotron X-ray findings of Fullwood N.J. and Meek K.M. [16], sample preparation for TEM inherently alters the sample. Glutaraldehyde fixation is associated with increased intermolecular spacings within the collagen fibril, while ethanol dehydration appears to reduce interfibrillar spacing [16]. Resin infiltration and polymerization can result in shrinkage of all of the spacings and loss of degree of order of the collagen molecules [16]. Post-fixation with osmium tetroxide may also affect packing of the collagen molecules [16]. Akhtar S. also confirmed that different processing techniques can produce variations in collagen fibril diameter and spacing [17].

Transmission electron microscopy was performed using the model TECNAI 20–120 KV BioTwin equipped with an Olympus MegaView TEM CCD camera system (FEI Co., Eindhoven, The Netherlands).

The study and publishing of the study results were approved by the Local Ethics Committee for Scientific Research of the Clinical Institute for Ophthalmological Emergencies “Prof. Dr. Mircea Olteanu” (Nr. 413/28 January 2026). Following the analysis of the submitted study documents regarding patient consent, pre- and post-surgical documentation, slit-lamp images, and the histopathological protocol and images from light microscopy and transmission electron microscopy (courtesy of paper’s author Gherghiceanu Mihaela, Victor Babeș National Institute for Pathology, Bucharest, Romania), the aforementioned Local Ethics Committee approved the publication of this study. This paper is part of doctoral PhD TEM research on collagen lamellae corneal ultrastructure.

## 3. Results

### 3.1. Corneal Ultrastructure in the Hematocornea DALK Transplant Graft (TEM)

**Figure 4:**
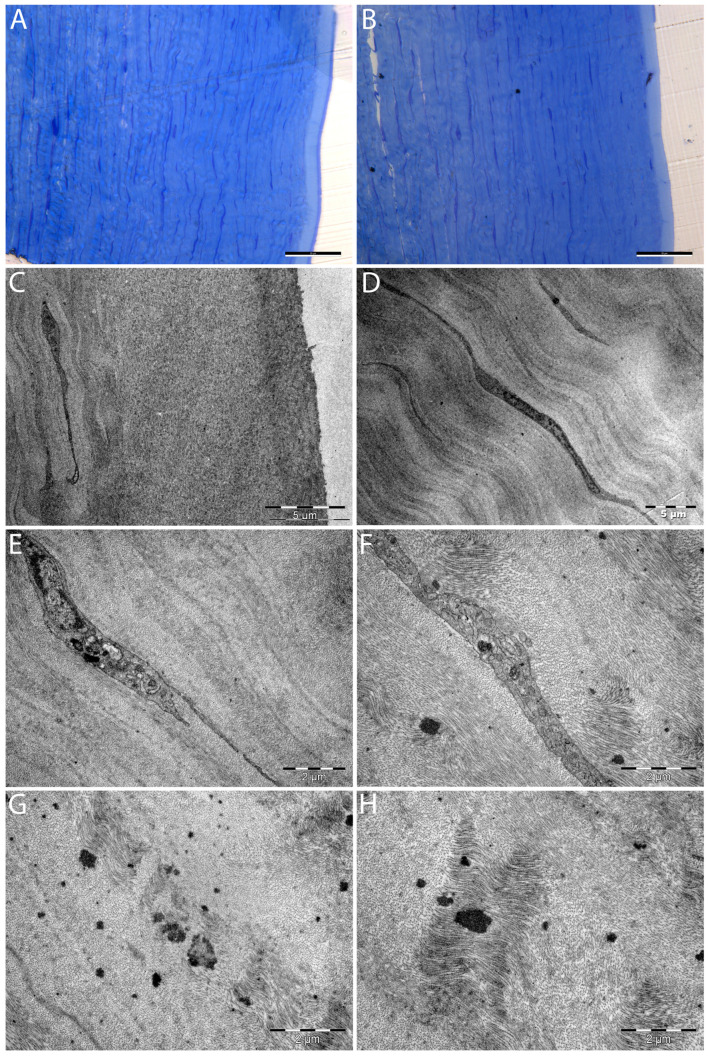
Corneal ultrastructure in the hematocornea DALK transplant graft. (**A**,**B**) Light microscopy. Scale bars: A and B 50 µm. Right: Epithelium and Bowman’s layer. Left: Stromal collagen lamellae (toluidine blue stain) with wave-like patterns. Leica DFC7000T camera. (**C**–**H**) Transmission electron microscopy. Scale bars C and D 5 µm, E to H 2 µm. (**C**) Transition from epithelium to anterior stroma. (**D**) The crumpled lamellar structure that can elongate to accommodate tensional forces (Meek K.M. et. al. [14]). Keratocyte between lamellae. (**E**) Cross-graining lamellae disposition: each layer is perpendicular to adjacent layers like plywood. Keratocyte with electron-dense material in phagosome. (**F**) Varied lamellae patterns (posterior stroma). Electron-dense hem inclusions adjacent to and inside of keratocyte. (**G**,**H**) Lamellae appear disorganized near electron-dense hem deposits, with interruption of the wavy lamellae pattern. FEI TECNAI BioTwin transmission electron microscope, Olympus MegaView CCD.

Corneal ultrastructure in the hematocornea DALK transplant graft (TEM) (Figure 4).

### 3.2. Cellular Response to Presence of Electron-Dense Hem Material

**Figure 5:**
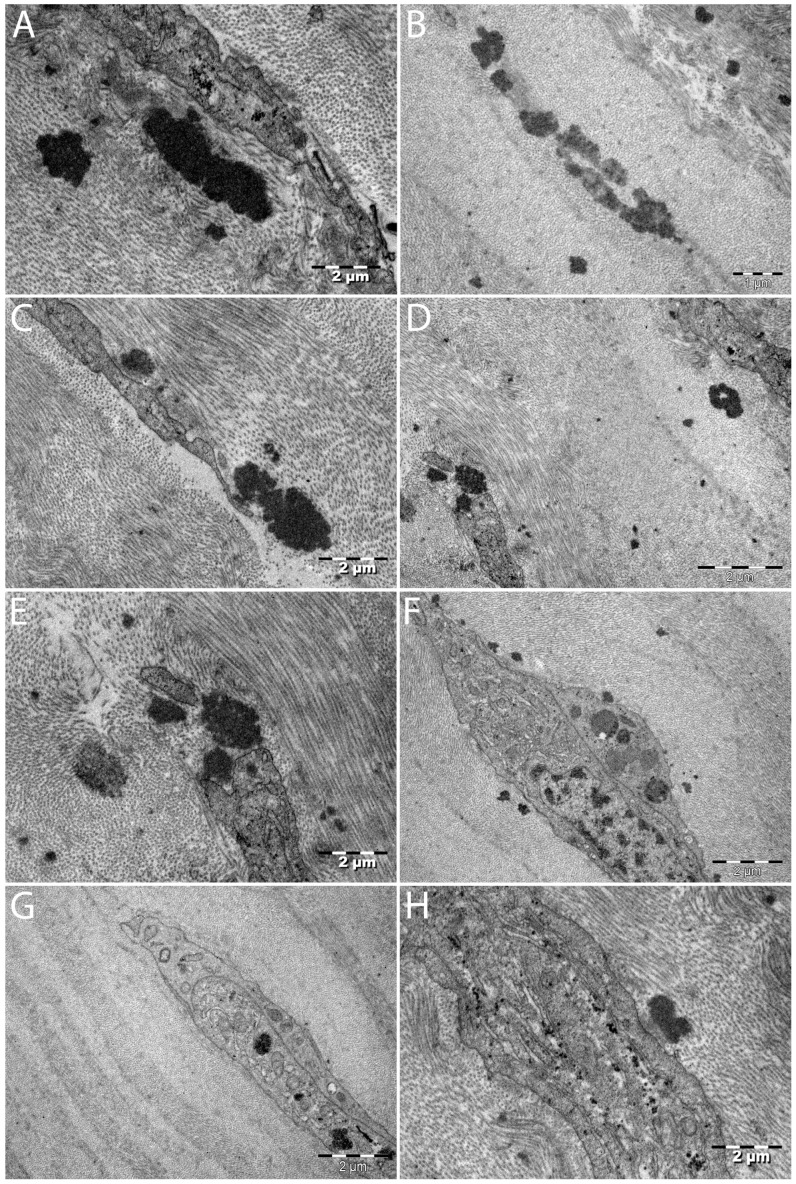
Cellular response to presence of electron-dense hem material. (**A**–**H**) Transmission electron microscopy. Scale bars: A and C to H 2µm; B 1 µm (**A**,**B**) Disorganized collagen lamellae near electron-dense hem agglomerations. (**C**–**E**) Cellular degeneration. (**C**) Degraded keratocyte with loss of organelle contours near electron-dense hem deposit. The cytoplasm contains electron-dense granules. Amorphous extracellular matrix may have replaced lamellae. (**D**,**E**) Numerous electron-dense deposits. (**E**) Burst-cell aspect at electron-dense hem deposit (loss of membrane integrity and organelle details). (**F**–**H**) Cellular adaptation. (**F**) Larger keratocyte (vs. Figure 4E) presenting electron-dense material in numerous vacuoles and a large phagosome. (**G**) Keratocyte with phagosome containing electron-dense material. (**H**) Phagocytic apparatus with numerous tiny electron-dense granules. FEI TECNAI BioTwin transmission electron microscope, Olympus MegaView CCD.

Cellular response to presence of electron-dense hem material (Figure 5).

## 4. Discussion

To our knowledge, there is a lack of more recent transmission electron microscopy (TEM) studies in hematocornea since the landmark 1985 study of McDonnell, P. J., Green, W. R., Stevens, R. E., Bargeron, C. B., and Riquelme, J. L. [10]. To our knowledge, our study may offer one of the first analyses of transplanted corneal tissue via TEM. While findings are hereby discussed at large, we caution that our study presents several limitations, such as a single-case design and absence of control tissue. We also consider the lack of comparative literature newer than 1985 as a limitation. This study may be novel as one of the first ultrastructural descriptions of graft hematocornea in DALK.

### 4.1. Common Findings with McDonnel et al. [10]

This study found many common elements with the TEM description of hematocornea provided by McDonnell, P.J. et al. [10]. Free hemoglobin aggregates presented as scattered electron-dense deposits throughout the stroma [10] (similar to Figure 4G,H and Figure 5A,B in our study). Regarding cellular aspects, both our study and McDonnel P.J. et al. described keratocytes, which appeared degenerated with loss of cell membrane and organelle details (Figures 5 and 6 from McDonnell P.J. [10] are similar to Figure 5 C–E in our study) or exhibiting cellular destruction according to McDonnell P.J. et al. [10].

### 4.2. Common Findings with Yoshimura M. et al. [12]

Another study in which findings share commonality and offer a comparison is that by Yoshimura M. et al. [12], which explored a corneal sample suffering from intracorneal hemorrhage in interstitial keratitis due to Hansen’s disease [12]. Like Yoshimura M. et al., we have noted disarrangement in the lamellar structure, with electron-dense granules between lamellae [12] and cells presenting abnormalities, such as large vacuoles in the cytoplasm [12].

### 4.3. Differences from Yoshimura M. et al. [12] and McDonnel et al. [10]

Yoshimura M. et al. noted finding parenchymal cells with abundant granules in their cytoplasm and regarded them as macrophages (Figure 4 from Yoshimura M. et al.) [12]. The authors described neovascular features (in infectious keratitis context), which could have aided macrophage infiltration of the corneal stroma [12]. In our study, we consider Figure 5F to present a cell with features, like Yoshimura et al. described, that appear to suggest a degree of similarity to a macrophage. Several vacuoles may be present in the top–middle of the cell, and a large phagosome with electron-dense material appears present towards the bottom–middle of the cell. However, in our study, this cell still retains a keratocyte cellular shape (different from Yoshimura M. et al. with macrophage shape); it is positioned between collagen lamellae like a keratocyte, and no nearby neovascular elements were found. Figure 4E and Figure 5G may present each a keratocyte with a phagosome. Figure 5H may present a phagocytic apparatus containing numerous tiny electron-dense granules. As such, in the case of our study, we can at most hypothesize, with caution, that we may have observed some cellular adaptations in the clearance of hem material. We caution that our study has several limitations, such as the single case design and lack of control tissue. Furthermore, we recommend that more study specimens or TEM studies are required to more accurately ascertain cellular features and types.

McDonnel P.J. et al.’s larger study of 11 hematocornea cases compared findings with the previous Yoshimura M. et al. study [10,12]. Concerning clearance of hemoglobin, McDonnel, P.J. et al. found only degraded keratocyte aspects without the presence of macrophages [10]. These correspond to the cellular degeneration aspects in this study (Figure 5C–E). McDonnel, P.J. et al. described the findings of Yoshimura M. et al. of cellular adaptations, such as vacuoles as well as the presence of macrophages, as a special case linked to interstitial keratitis and corneal neovascularization [10,12]. Specifically, according to the larger and established McDonnel P.J. et al. study, the primary clearance mechanism of hem products is not cellular ingurgitation of the material but instead diffusion towards limbal circulation and within the anterior chamber [10]. Thus, there is no correspondence between the possibility of cellular adaptations, as hypothesized here (Figure 5F–H), and the larger 11-case McDonnel P.J. et al. study.

### 4.4. Further Research on Hemoglobin Clearance and Cellular Adaptation

Macropinocytosis?

Upon further literature research on hemoglobin clearance, we observed distant potential connection with our study. The most specialized cellular system for extracellular hemoglobin clearance is the haptoglobin–hemoglobin clearance mechanism through macrophage scavenger receptor CD163 [18]. CD163 liver macrophages detoxify excess hemoglobin from erythrocyte lysis [19,20]. Interestingly, in a recent study, Zurawska, G. et al. observed that in sickle cell disease, a pathological condition where there is an excessive turnover of erythrocytes and excess of hem products to detoxify, extracellular hemoglobin can be uptaken by cells not normally associated with hemoglobin clearance: liver sinusoidal cells (LSECs) via macropinocytosis. According to Zurawska G. et al., control samples had hemoglobin only ingurgitated by specialized macrophages, while in sickle cell disease LSECs also started taking up hem products via macropinocytosis. These LSECs presented an electron-dense microscopy aspect that was not found on control tissue [20]. Furthermore, the authors confirmed via markers that hem processing by LSECs leads to cell senescence (degradation) and suggested that intracellular accumulation of Hemoglobin-S can induce cellular toxicity for LSECs [20].

Macropinocytosis can be upregulated by corneal epithelial cells [21] in a post-traumatic iatrogenic context after trephine corneal injury [21] (our hematocornea patient had previous DALK surgery in which a trephine was used to start DALK stromal dissection). According to Peng H. et al., macropinocytosis may play a role in various stromal corneal perturbations, such as stromal swelling [22]. The stem-cell-enriched limbal epithelium can carry out both autophagy and macropinocytosis [22]. Peng, H. et al. demonstrated that the macropinocytosis process of corneal epithelial cells is linked via MIR103-MIR107 and that loss of MIR103-MIR107 leads to dysregulation and the creation of large cytoplasmatic vacuoles, which have an autophagic effect on the cell [22]. Corneal epithelial cells specifically lack MIR103-MIR107, implying that regulation against massive or dysregulated macropinocytosis is absent [22].

With regards to keratocyte cellular adaptability, it is known that under release of growth factors and pro-inflammatory cytokines, keratocytes can be adapted into migratory fibroblasts and/or fibrotic myofibroblast phenotype [23,24]. According to Sato, T. et al., while keratocytes are not professional phagocytosis cells like macrophages, they do possess latent capability for phagocytosis [25]. Under pathological conditions, keratocytes activate, undergo cell division, increase in size, and manifest increased phagocytic activity [25,26,27,28,29]. Furthermore, according to findings from Chakravarti S., keratocytes express gene characteristics of macrophages, such as genes encoding mannose receptors, lipocalins, chemokines, interleukin-1 receptor, and major histocompatibility complex class I antigens [29]. This mechanism of keratocyte plasticity [29] serves as an innate defense against corneal infection or injury as the cornea is an avascular tissue devoid of vascular pathways for bringing in professional phagocytosis cells [25,29].

These potential distant connections may provide interest for the observed electron-dense material inside of keratocytes, cells not normally associated with hemoglobin ingestion and clearance. Macropinocytosis may mechanistically explain the ingestion of electron-dense granules, and dysfunction in the macropinocytosis process may lead to cell degeneration. We state that these aspects have not been confirmed or studied at large in hematocornea and that our study presents important limitations (single-case, no control tissue). We recommend further studies based on this hypothesis.

### 4.5. Two Cellular Aspects in Hematocornea: Adaptation and Degradation

#### 4.5.1. Cellular Adaptation

As previously stated, our study hypothesizes, with caution as a single-sample TEM study with no control tissue, that we may have observed some cellular adaptations to the clearance of hem material (Figure 5F–H). This description is not correspondent to the previous and larger McDonnel P.J. et al. study [10] and may have been made possible in part due to faster sample retrieval time to TEM microscopy (direct from the operating room) and higher-resolution TEM technology, unavailable in previous studies. We suggest at most simple cellular adaptation, as cells in our study retained a keratocyte form and general aspect. Ingurgitated electron-dense material was found apparently free in the cytoplasm in very small granules, included in larger vacuoles, and as even larger deposits included in phagosomes (Figure 5F). As discussed previously in Section 4.4, macropinocytosis may mechanistically explain the ingestion of electron-dense material. We lack a direct comparison study for these findings, as Yoshimura et al.’s sample [12] had infectious interstitial keratitis and neovascularization (which can explain the presence of macrophages), and samples from McDonnel P.J. et al. only had intracytoplasmatically electron-dense deposits [10]. As such, we consider these findings as potentially novel and state that further TEM studies on more samples are required.

#### 4.5.2. Cellular Degradation

Cellular degradation has been observed in our study (Figure 5C–E). This finding is in line with both McDonnel et al. [10] and Yoshimura M. et al. [12]. In our case, once the hematocornea was established, clearance of the hemoglobin molecules was dysfunctional. We observed altered collagen lamellae (Figure 4G–H and Figure 5A,B) and keratocytes (Figure 5D–E). Interestingly, we also observed a burst-cell keratocyte aspect surrounding an electron-dense hem deposit (Figure 5E). Numerous keratocytes close to electron-dense deposits presented degraded aspects, such as loss of the cell membrane and organelle details (Figures 5 and 6 from McDonnell P.J. [10] are similar to Figure 5C–E in our study). Overall, keratocytes suffered in our hematocornea sample, and hemoglobin clearance seems to be dysfunctional.

#### 4.5.3. Cellular Analysis and Study Limitations

The simultaneous presence of viable adapted cellular aspects as well as degraded cells in the same sample is in line with our study of images from different stromal areas. Transmission electron microscopy analysis is highly dependent on the time from sampling to fixation, with every minute counting. In our study, fixation was started immediately from the operating room through immersion in glutaraldehyde solution transport medium and quickly completed once the sample arrived at the TEM. We caution that even so, loss of cell vitality cannot be avoided. Transport and fixation of TEM samples stop cell vitality and can induce fixation artifacts. Specifically, due to sample dehydration during preparation, fixation artifacts can include modifications in inter-collagen-lamellae spaces, as described by Fullwood N.J. and Meek K.M [16] and Akhtar S. [17]. Osmium tetroxide can oxidize alpha amino acids and cause loss of proteins [16].

### 4.6. Alteration of the Collagen Lamellae

The lamellar corneal ultrastructure seemed altered in close proximity to electron-dense hemoglobin products. Gaps in the lamellar structure were noticeable with replacement extracellular matrix (Figure 4G–H and Figure 5A–E). Altered lamellar were present near larger electron-dense hem deposits. Such changes in the lamellar structure were also described by Yoshimura M. et al. in intracorneal hemorrhage in interstitial keratitis due to Hansen’s disease [12].

### 4.7. Loss of the DALK Graft

This microscopical alteration of the corneal ultrastructure could explain the loss of two corneal properties: loss of corneal elasticity and loss of corneal transparency. Due to interruptions in the lamellar structure as well as differently electrically charged hem molecules, the collagen fibril response to external forces could be altered, resulting in total loss of corneal elasticity. Our sample was so rigid that it posed difficulty upon cutting for TEM analysis. Transparency degradation is probable due to hem product deposits, which are opaque. Meek K.M. et al. also state that alterations in the gaps and disposition of collagen lamellae lead to chaotic light scattering, decreasing corneal transparency and optical performance [14]. Thus, the DALK graft was altered, and re-transplant via penetrative keratoplasty was necessary.

### 4.8. Clinical Context of the Case

#### 4.8.1. Preoperative Surgical Risk Assessment

According to Kodavoor et al., breach of Descemet’s membrane is the most frequent complication associated with DALK (6.54–6.6% of cases) [2,3]. Literature recommendations are to continue DALK surgery, as postoperative results even with breach of Descemet’s membrane are superior versus conversion to penetrative keratoplasty [4,5]. Our patient initially presented with post-traumatic secondary corneal ectasia, and DALK was chosen due to corneal thinness and risk of further ocular complications.

Preoperative health assessment of the corneal tissue and risk stratification benefit from novel technology, such as the latest-generation combined placido disc and high-resolution anterior segment optical coherence (AS-OCT) tomography and topography [30,31]. AS-OCT can also be enhanced with novel neural network screening [30]. In our case, screening with AS-OCT (MS-39, CSO, Italy) was performed (Figure 1). AS-OCT screening raised suspicion of Descemet’s membrane involvement. Another screening innovation is represented by in vivo confocal microscopy (IVCM) [32]. IVCM is a contact method and was available as HRT-3, Heidelberg Retina Tomograph 3/Rostock Cornea Module, Heidelberg Engineering, Heidelberg, Germany. IVCM can diagnose conditions such as corneal dystrophies and latent viral or fungal infection that are otherwise subclinical and escape traditional examination techniques [32,33]. Furthermore, IVCM can directly visualize endothelial and Descemet’s aspects and is especially useful in dystrophy diagnosis [34]. IVCM screening could have been of aid for direct Descemet visualization.

#### 4.8.2. Surgical and Postoperative Case Particularities

Considering case characteristics (Figure 1), DALK surgery proceeded. Unfortunately, despite best practice, intraoperative breach of Descemet’s membrane occurred. To restore the anterior chamber, several rescue techniques were employed, such as lowering intraocular pressure via paracentesis [7] and use of intracameral air tamponade to restore the anterior chamber [7]. Air tamponade produced modest results, enough to complete the placing of the DALK graft. To prevent pupillary block, an iridectomy was performed; however, this maneuver produced a severe and unexpected intracameral hemorrhage, which was difficult to control. Our approach was conservative, finishing DALK surgery in the best possible conditions and applying medical postoperative hemostatic treatment. Further surgical intervention was deferred to 1 month postoperative when intraocular inflammation and hemorrhage subsided.

We mention that in the case of an intact Descemet’s membrane and neovascular causes, Scorcia, V. et al. described a technique of stromal peeling and second DALK that can also resolve hematocornea [35]. In our case, re-transplantation via PK was chosen as a surgical solution due to previous breach of Descemet’s membrane and visualized anterior chamber adherences to the endothelium (Figure 2C). During PK, we found intraocular blood clots and adherences occupying the anterior chamber. Careful dissection and excision of adherences were performed, made difficult by bleeding from iris tissue (Figure 3C). The IOL was found displaced upwards and entrapped within adherences. The IOL was explanted to prevent pupillary block and other complications. Reintervention via PK was successful in restoring anterior chamber architecture and a clear visual axis (Figure 3D,E). At 4-month follow-up, no inflammation or hemorrhage was present, and BCVA improved to 20/100–20/200 (patient aphakic). We could not confirm preexisting conditions for the intracameral hemorrhage, such as coagulopathy or neovascularization in a post-traumatic context [36]. We caution that in a post-traumatic context, DALK surgery may present higher risks than initially expected.

## 5. Conclusions

We have observed that hematocornea in DALK corneal transplant altered the corneal collagen ultrastructure in the immediate proximity. Two keratocyte cellular aspects were observed: adaptation and degradation. Adapted cells ingurgitated large amounts of electron-dense hem material intracytoplasmatically as small granules, as larger granules in vacuoles, and as even large deposits in phagosomes. Macropinocytosis may mechanistically explain the ingestion of electron-dense granules, and dysfunction in the macropinocytosis process may lead to cell degeneration. Degenerated cells lost organelle details and cellular membrane integrity. These microscopic alterations affected corneal transparency and elasticity in the DALK graft. These observations may be novel, as, to our knowledge, there is a lack of TEM studies of DALK corneal transplant grafts. We recommend anterior segment imagistic screening before performing DALK in post-traumatic contexts, as surgery can be higher-risk than first evaluated. Furthermore, if intracameral hemorrhage occurs, we caution that hematocornea induction could be more likely, and specific adaptations to surgical technique and treatment may be necessary.

## Figures and Tables

**Table 1:** Complete sample preparation protocol.

Protocol	Specifications
Sample fixation using glutaraldehyde 4%	4–12 h
Resample with sodium cacodylate 0.1 M ^1^	4 °C, 2 × 10 min
Post-fixation with osmium tetroxide 0.1 M in sodium cacodylate 0.2 M	4 °C, 30 min
Sample suspension in osmium tetroxide 0.1 M	4 °C, 2 × 10 min
Dehydration in ethylic alcohol 30°	4 °C, 15 min
Dehydration in ethylic alcohol 50°	4 °C, 15 min
Dehydration in ethylic alcohol 70°	4 °C, 15 min
Dehydration in ethylic alcohol 90°	4 °C, 15 min
Dehydration in ethylic alcohol 96°	4 °C, 15 min
Dehydration in ethylic alcohol 100°	Room temperature, 2 × 15 min
Propylene oxide	Room temperature, 3 × 10 min
Bath I: propylene oxide/epoxy resin (2/1)	Room temperature, 2 h
Bath II: propylene oxide/epoxy resin (1/2)	Room temperature, overnight
Bath III: epoxy resin (Agar 100)	Room temperature, 3 h
Epoxy resin polymerization	60 °C, 48 h

^1^ M—molar. Courtesy of paper’s author Mihaela Gherghiceanu, Victor Babeș National Institute for Pathology, Bucharest, Romania.

## Data Availability

All data from the study has been included in the paper.

## Abbreviations

The following abbreviations are used in this manuscript:

DALK	Deep anterior lamellar keratoplasty
TEM	Transmission electron microscopy
AS-OCT	Anterior pole optical coherence tomography
AC	Anterior chamber
mm	Millimeter
µm	Micron
M	Molar
°C	Degree Celsius
IVCM	In vivo confocal microscopy
LSECs	Liver sinusoidal cells
HbS	Hemoglobin S (from sickle cell disease)
